# Impact of insertion into the left internal jugular vein in chemoport-associated infections: a retrospective single-center study of 1690 cases

**DOI:** 10.1038/s41598-024-59749-2

**Published:** 2024-04-18

**Authors:** Gwon-Min Kim, Seunghwan Song, Do Young Kim, Soo Han Kim, Chung Won Lee, Miju Bae, Jong Won Kim, Up Huh

**Affiliations:** 1 Medical Research Institute, Pusan National University, Busan, Republic of Korea; 2 Department of Thoracic and Cardiovascular Surgery, Pusan National University School of Medicine, Biomedical Research Institute, Pusan National University Hospital, Busan, Republic of Korea; 3 Department of Hematology-Oncology, Pusan National University School of Medicine, Biomedical Research Institute, Pusan National University Hospital, Busan, Republic of Korea; 4 Division of Respiratory, Department of Internal Medicine, Pusan National University School of Medicine, Biomedical Research Institute, Pusan National University Hospital, Busan, Republic of Korea

**Keywords:** Chemoport, Infection, Internal jugular vein, Cancer, Oncology

## Abstract

We analyzed chemoport insertion procedures to evaluate infectious morbidity and factors causing infection. This single-center retrospective study included 1690 cases of chemoport implantation between January 2017 and December 2020. Overall, chemoports were inserted in 1582 patients. The average duration of chemoport use was 481 days (range 1–1794, median 309). Infections occurred in 80 cases (4.7%), with 0.098 per 1000 catheter-days. Among the 80 cases in which chemoports were removed because of suspected infection, bacteria were identified in 48 (60%). Significantly more cases of left internal jugular vein punctures were noted in the infected group (15 [18.8%] vs. 147 [9.1%]; p = 0.004). Pulmonary embolism was significantly different between the infection groups (3 [3.8%] vs. 19 (1.2%), p = 0.048). The hazard ratio was 2.259 (95% confidence interval [CI] 1.288–3.962) for the left internal jugular vein, 3.393 (95% CI 1.069–10.765) for pulmonary embolism, and 0.488 (95% CI 0.244–0.977) for chronic obstructive pulmonary disease. Using the right internal jugular vein rather than the left internal jugular vein when performing chemoport insertion might reduce subsequent infections.

## Introduction

Since the introduction of the port system by Niederhuber et al.^1^, the use of chemoports in patients undergoing chemotherapy has increased considerably. The use of chemoports has enabled the safe administration of high-vesicant chemotherapeutic drugs. In addition, it can be used for parenteral nutrition, transfusion of blood products, antibiotics, intravenous fluid administration, and intravenous sampling, and has the advantage of avoiding frequent cannulation^2,3^. Another advantage of the chemoport is that it requires minimal care and maintenance to remain functional when not in use.

However, because chemoports act as foreign substances in the body, serious complications, such as infection, sepsis, venous thrombosis, mechanical dysfunction, catheter disconnection, and embolism may occur^4^. Infection is one of the most common complications associated with chemoport use. Infection is a serious complication that may require early chemoport removal, and treatment may delay chemotherapy. Approximately 4.8% chemoport-associated infections have been reported in the literature^5^. Infections that occur during treatment can result in prolonged hospitalization and higher healthcare costs.

Patients with cancer have a weakened immune system during the course of the disease; therefore, preventing infection is essential for caring for patients with cancer. We analyzed chemoport insertion procedures performed in a single institute for 4 years to evaluate infectious morbidity and the factors causing infection.

## Patients and methods

This study was retrospectively conducted on 1690 cases of chemoport implantation in patients who underwent chemoport insertion for chemotherapy at a single institute in Busan, Republic of Korea, between January 2017 and December 2020. Based on the patients’ medical records, we investigated their sex, age, cancer type, use of antithrombotic drugs, and basic medical history. Information related to chemoport insertion, duration of use, vein used for insertion, location of the catheter tip, angle of catheter entry, and other complications such as infection and blood clots were investigated.

All procedures involving human participants performed in this study were in accordance with the ethical standards of the institutional and/or national research committee and with the 1964 Helsinki Declaration and its later amendments or comparable ethical standards. This study was approved by the Pusan National University Hospital Institutional Review Board (IRB No. 2307-007-128). Before all procedures, the potential risks and benefits were explained in detail to the patients, and written informed consent was obtained.

Two cardiovascular surgeons inserted the chemoports. Contraindications included a platelet count < 50,000, an absolute neutrophil count < 500/mm^3^ and a prothrombin time international normalized ratio > 1.5. For prophylaxis, first-generation cephalosporin was intravenously administered once immediately before surgery, and no additional antibiotics were used thereafter. All patients underwent chemoport insertion in the operating room under local anesthesia. In all patients, the internal jugular vein was punctured using the percutaneous Seldinger technique under ultrasound, a catheter was inserted, and the right internal jugular vein was preferentially used in all patients except for patients with right breast cancer. The left internal jugular vein was used when it was impossible to puncture the right internal jugular vein or in patients with right breast cancer. After insertion, the position of the distal tip of the catheter was confirmed using the C-arm. A pocket for port insertion was created using an electrocautery to create a 2 cm × 2 cm space under the skin through an incision of approximately 1.5–2 cm. After port insertion, the pocket was sutured subcutaneously and continuously using Vicryl 4-0 (Ethicon). Dermabond Advanced (Ethicon, Cincinnati, Ohio, USA) was applied to the skin. As Dermabond Advanced was used, no additional dressing was applied after the operation, and the wound was usually checked on an outpatient basis on postoperative day 7.

The chemoport, including a dressing, was primarily managed by a trained nurse. While using the chemoport, a skilled nurse changed the dressing once every 7 days but immediately if sweaty or dirty. If the port had not been used long, it was flushed every 4 weeks. The port was flushed with 10 mL of 0.9% saline and closed with 4–8 mL of heparinized saline (100 IU/mL) every 4 weeks after insertion and after access to prevent blockage.

Local infection is a case in which symptoms, such as feeling warm to the touch, redness, swelling, discharge or pus, long-lasting pain, and tenderness, occur at the site where the catheter passes through the subcutaneous layer, such as the chemoport insertion site, needling site, and chemoport to the internal jugular vein. Systemic infection was defined as fever, chills, leukocytosis, C-reactive protein elevation, and bacterial identification in blood culture, even in the absence of signs of local infection. In all cases, the chemoport was removed immediately after infection was strongly suspected, and empirical antibiotics and antibiotics appropriate for the bacteria found in the blood culture or wound culture were administered in consultation with the infectious medicine specialist. Empirical antibiotics used were vancomycin and vancomycin plus cefepime. Antibiotic treatment was administered for at least 2 weeks, and when bacteria were not identified at least twice in the blood and wound cultures, antibiotic treatment was discontinued in consultation with an infectious medicine specialist. If pus was present at the pocket or incision site where the chemoport was inserted, betadine soaking was performed without wound closure. If bacteria were not identified more than twice at the wound site, simple suturing was performed using nylon 3-0 on the wound.

Thrombosis was catheter-related, and deep vein thrombosis unrelated to the catheter was excluded. The catheter tip location was measured as the distance from the carina to the catheter tip, and the catheter angle was defined as the angle between the apex inserted into the port and the internal jugular vein and the catheter tip. We investigated whether there were differences according to the type or location of catheter insertion. A change in the position of the catheter was defined as a change in the position of the catheter tip during use, causing malfunction and requiring removal.

Statistical analyses were performed using the SPSS software (version 25.0; IBM Corp., Armonk, NY, USA). Data are presented as frequencies, proportions, and means ± standard deviations. All variables were used to compare the datasets of the infected and non-infected groups. Independent t-tests or Mann–Whitney U tests were used for continuous variables. The chi-square test was used for continuous variables. Pearson’s correlation coefficient and Fisher’s exact test were used to evaluate the degree of correlation between variables. We used propensity score matching (PSM) and a t-test to eliminate bias between groups. Cox hazard regression was used to estimate the risk ratio for sex, age, solid cancer, antiplatelet therapy, left internal jugular vein, catheter tip location, catheter angle, occlusion, catheter-related thrombosis, hypertension, diabetes, coronary artery occlusive disease, peripheral artery occlusive disease, cerebrovascular accident, chronic obstructive pulmonary disease (COPD), and renal insufficiency based on the infection status. Additionally, a Cox proportional hazards regression model was used to perform multivariate prognostic analyses for left internal jugular vein, catheter tip location, occlusion, malfunction, catheter-related thrombosis, catheter angle, and pulmonary embolism. Statistical significance was set at p < 0.05.

## Results

A total of 1690 chemoports were inserted in 1582 patients. In majority of cases (1582 cases), the procedure was performed without immunosuppression because the chemoport was performed prior to initiating anticancer treatment. Among the remaining 108 cases, there were 3 instances of reinsertion in 4 patients. In cases where reinsertion followed an infection, the port was first removed for infection treatment and then reinserted at least 1 month after no bacteria were identified or no signs of infection occurred. In cases where the port was reinserted due to recurrence, chemotherapy was not performed for at least 3 months. The average duration of chemoport use was 481 days (range 1–1794 days, median 309 days). Infections occurred in 80 cases (4.7%), with 0.098 per 1000 catheter days. No cases infected more than twice were observed. Among the 80 cases in which chemoports were removed because of suspected infection, bacteria were identified in 48 (60%). Among the 80 cases of infection, 34 cases had signs of systemic infection, and 48 cases had local signs of infection. In 2 cases, both local and systemic infection signs were observed. Notably, 48 cases of infection occurred during outpatient chemotherapy, with 3 cases showing systemic infection signs and 47 cases showing local infection signs. Additionally, 32 cases experienced infection during long-term hospitalization, with 31 exhibiting systemic infection signs and 1 showing local infection signs. The infection group comprised 16, 10, 7, 3, and 2 patients with breast, lung, stomach, colon, and thymic carcinoma, respectively. It also comprised two cases of bladder and pancreatic cancer, respectively. In addition, one patient each had cholangiocarcinoma, esophageal cancer, neurofibromatosis, ovarian cancer, peritoneal cancer, supraglottic cancer, thymic epithelial tumor, and thyroid cancer. In the infection group, the hematological malignancies were lymphoma in 11 patients, leukemia in 2, and multiple myeloma in 3. The characteristics of the patients are described in Table 1. No significant differences were observed between the infected and non-infected groups.

**Table 1:** Patient characteristics by differences between infection and non-infection.

	Overall (n = 1690)	Infection (n = 80)	Non-infection (n = 1610)	p
Male	956 (56.6)	46 (57.5)	910 (56.5)	0.863
Age (years)	63.38 ± 11.58	61.53 ± 12.66	63.47 ± 11.52	0.142
Solid cancer	1404 (83.1)	65 (81.3)	1339 (83.2)	0.655
Hypertension	634 (37.5)	30 (37.5)	604 (37.5)	0.998
Diabetes	429 (25.4)	16 (20.0)	413 (25.7)	0.257
Coronary artery occlusive disease	110 (6.5)	2 (2.5)	108 (6.7)	0.136
Peripheral artery occlusive disease	8 (0.5)	1 (1.3)	7 (0.4)	0.300
Cerebrovascular accident	97 (5.7)	4 (5.0)	93 (5.8)	0.771
COPD	110 (6.5)	9 (11.3)	101 (6.3)	0.078
Renal insufficiency	103 (6.1)	7 (8.8)	96 (6.0)	0.309
Antiplatelet	238 (14.1)	9 (11.3)	229 (14.2)	0.455

COPD: chronic obstructive pulmonary disease.

The identified bacterial species are listed in Table 2. *Pseudomonas aeruginosa*, *Klebsiella pneumoniae*, and *Enterococcus faecium* were also identified in one patient with *Candida albicans*. *Enterobacter cloacae* and *Enterococcus faecium* were also identified in one patient with *Candida tropicalis*. In the infection group, there were four in-hospital deaths due to sepsis, and all bacteria were identified only by blood culture. The causative organisms include *Staphylococcus epidermidis* (methicillin-resistant *Staphylococcus epidermidis*), *Candida parapsilosis*, *Candida glabrata*, and *Enterococcus faecium*.

**Table 2:** Types of pathogens identified in the infection group.

Types of pathogens	N = 80
Not identified	32
Identified	48
Gram positive	25
*Staphylococcus* spp.	19 (12)
*Staphylococcus epidermidis*	9 (6)
*Staphylococcus aureus*	5 (4)
*Staphylococcus caprae*	2 (1)
*Staphylococcus capitis*	1
*Staphylococcus hominis*	1 (1)
*Staphylococcus lugdunensis*	1
*Corynebacterium striatum (G* +*)*	2
*Bacillus species (G* +*)*	1
*Corynebacterium tuberculostearicum (G* +*)*	1
*Enterococcus faecium(G* +*)*	1
* Streptococcus parasanguinis (G* +*)*	1
Gram negative	12
* Enterobacter cloacae* complex	2
* Escherichia coli*	2
* Pseudomonas aeruginosa*	2
* Stenotrophomonas maltophilia*	2
* Acinetobacter calcoaceticus-baumannii* complex	1
* Achromobacter xylosoxidans*	1
* Klebsiella pneumonia*	1
* Serratia marcescens*	1
Candida spp.	11
* Candida albicans*	3
* Candida glabrata*	2
* Candida parapsilosis*	2
* Candida tropicalis*	4

(*Methicillin-resistant staphylococcus spp.).*

The results are described in Table 3. The duration of use was significantly shorter in the infection group than in the non-infection group (230.81 ± 221.23 days vs. 465.39 ± 387.62 days; p < 0.001). There were significantly more cases of left internal jugular vein punctures in the infected group (15 [18.8%] vs. 147 [9.1%]; p = 0.004). No difference was observed in catheter-related thrombosis between the infection and non-infection groups (1 [1.3%] vs. 23 [1.4%]; p = 0.895); however, pulmonary embolism was significantly different in the infection group (3 (3.8%) vs. 19 (1.2%); p = 0.048). Table 4 shows the results of the transformations using PSM. The infected group had a significantly shorter duration of catheter use compared to the non-infected group (244.34 ± 228.00 days vs. 447.68 ± 344.70 days; p < 0.001). Moreover, the infected group had significantly more left internal jugular vein punctures (11[16.2%] vs 3[4.4%]; p = 0.024).

**Table 3:** Factors associated with chemoport.

	Overall (n = 1690)	Infection (n = 80)	Non-infection (n = 1610)	p
Duration of use (days)	454.28 ± 384.58	230.81 ± 221.23	465.39 ± 387.62	< 0.001
Left internal jugular vein	162 (9.6)	15 (18.8)	147 (9.1)	0.004
Catheter-related thrombosis	24 (1.4)	1 (1.3)	23 (1.4)	0.895
Occlusion, malfunction	49 (2.9)	2 (2.5)	47 (2.9)	0.827
Position change of catheter tip	11 (0.7)	0 (0.0)	11 (0.7)	0.458
Pulmonary embolism	22 (1.3)	3 (3.8)	19 (1.2)	0.048
Catheter tip location (cm)	28.52 ± 17.35	29.38 ± 18.48	28.48 ± 17.29	0.651
Catheter angle	52.50 ± 12.45	54.03 ± 11.63	52.42 ± 12.49	0.262

The catheter tip location was measured as the distance from the carina to the catheter tip, and the catheter angle was defined as the angle between the apex inserted into the port and the internal jugular vein and the catheter tip.

**Table 4:** Factors associated with chemoport using propensity score matching.

	Overall (n = 136)	Infection (n = 68)	Non-infection (n = 68)	p
Duration of use (days)	346.01 ± 308.52	244.34 ± 228.00	447.68 ± 344.70	< 0.001
Left internal jugular vein	14 (10.3)	11 (16.2)	3 (4.4)	0.024
Catheter-related thrombosis	2 (1.5)	1 (1.5)	1 (1.5)	0.999
Occlusion, malfunction	6 (4.4)	2 (2.9)	4 (5.9)	0.404
Position change of catheter tip	1 (0.7)	0 (0.0)	1 (0.7)	0.316
Pulmonary embolism	4 (2.9)	3 (4.4)	1 (1.5)	0.310
Catheter tip location (cm)	27.29 ± 15.89	28.53 ± 18.17	26.04 ± 13.25	0.364
Catheter angle	52.38 ± 13.21	54.09 ± 10.87	50.68 ± 15.09	0.133

The catheter tip location was measured as the distance from the carina to the catheter tip, and the catheter angle was defined as the angle between the apex inserted into the port and the internal jugular vein and the catheter tip.

**Figure 1:**
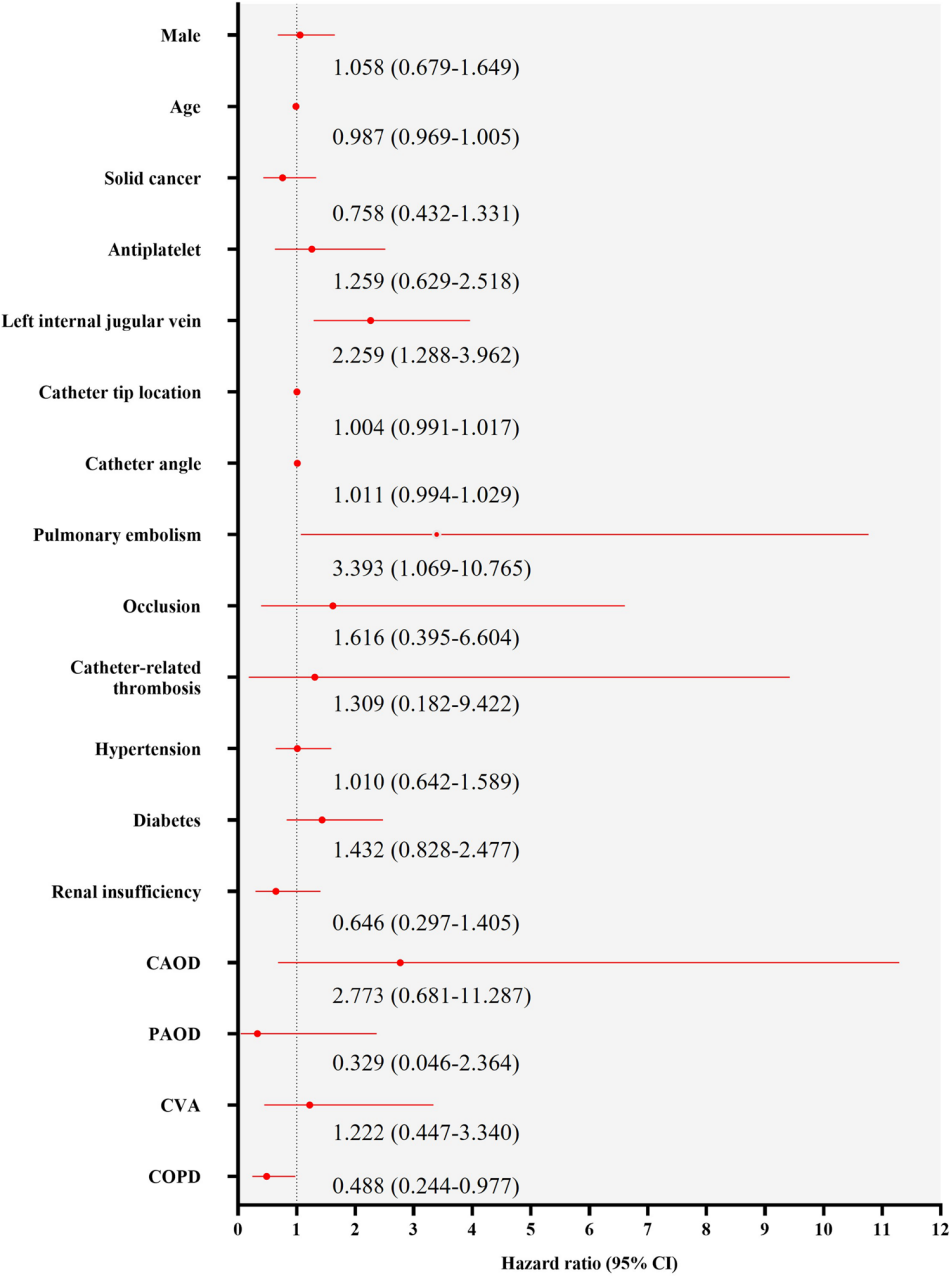
Cox proportional hazards regression analysis for the cause of infection. CAOD, coronary artery occlusive disease; PAOD, peripheral artery occlusive disease; CVA, cerebrovascular accident; COPD, chronic obstructive pulmonary disease; CI, confidence interval.

Figure 1 shows the hazard ratios for disease incidence based on infection status and catheter use duration. The hazard ratio was 2.259 (95% confidence interval [CI] 1.288–3.962) for the left internal jugular vein, 3.393 (95% CI 1.069–10.765) for pulmonary embolism, and 0.488 (95% CI 0.244–0.977) for COPD, indicating that patients who developed an infection had a 2.259-, 3.393-, and 0.488-times higher risk, respectively. The hazard ratios for the transformations using PSM are shown in Table 5. The multivariate prognostic analysis for the left internal jugular vein complications yielded a hazard ratio of 3.120 (95% confidence interval [CI] 1.498–6.498).

**Table 5:** Cox proportional hazards regression analysis for the cause of infection using propensity score matching.

Variables	HR (95% CI)
Left internal jugular vein	3.120 (1.498–6.498)
Catheter tip location (cm)	0.997 (0.981–1.014)
Occlusion, malfunction	2.361 (0.550–10.128)
Catheter-related thrombosis	0.812 (0.101–6.530)
Catheter angle	1.013 (0.995–1.031)
Pulmonary embolism	1.689 (0.506–5.638)

HR, hazard ratio; CI, confidence interval.

## Discussion

Infection is the most common and feared complication in patients undergoing chemotherapy via a chemoport. However, completely implantable venous access ports have a lower risk of infection and last longer than other intravenous devices^6^. Approximately 3–10% of patients experience infection associated with the chemoport, which is the most frequent cause of chemoport removal^6–11^. In our study, the incidence of infection was 4.7%, which is not significantly different from that reported in previous studies. However, the infection rate in this study (0.098 per 1000 catheter days) was lower than that of previous studies (range 0.11–0.37 per 1000 catheter-days)^12^. Once a catheter-associated infection is diagnosed, the chemoport must be removed, broad-spectrum antibiotic therapy is administered, and chemotherapy is deferred. Prevention of catheter-associated infections is crucial, with strict adherence to universal precautions for asepsis, such as hand washing and aseptic techniques^5^. Nurses accessing the chemoport must be trained to wear face masks, caps, and sterile gloves^12^. The needle should be disinfected with alcohol-based chlorhexidine or povidone-iodine each time it is inserted^13^. The Huber needle should be replaced every week if vascular access is continuously maintained^6^. Patients should be educated about the potential risk of catheter-related infections and informed that only staff trained in aseptic techniques should have access to the device.

In our study, port infection occurred on average 230.81 ± 221.23 days after implantation. These results are more likely caused by infection due to the long-term use of chemoports or reduced immunity of patients associated with long-term chemotherapy, rather than immediate postoperative infection. Therefore, most bacteria identified in this study were skin flora, non-glucose-fermentative gram-negative bacilli, and Candida species. Staphylococcus, the predominant species found on human skin, is the most common cause of catheter-associated infections^14,15^. To prevent and reduce infection by skin flora and non-glucose fermentative gram-negative bacilli, centralized management and maintenance standards for port insertion sites, especially needling sites, must be established and thoroughly managed^15^. *Candida* infections usually occur in immunocompromised hosts^16^. *Staphylococcus* and *Candida* species bind well to the host proteins and attach better to silicone catheters. Total parenteral nutrition can easily cause fungal infections, and ports may not be suitable for delivering it to cancer patients^6^.

The use rate of the left internal jugular vein was higher in the infected group (15 [18.8%] vs. 147 [9.1%]; p = 0.004). In addition, the hazard ratio for the left internal jugular vein was 2.259 (95% CI 1.288–3.962). We could not find a reference that could prove the difference in the risk of infection between the right and left sides of the central venous catheter. Further investigation through additional literature revealed a study that reported no difference in the occurrence of complications between patients who had a port inserted on the left and those who had a port inserted on the right after the right port was removed^17^. In addition, another study found more frequent infections with right-sided insertion within the first 2 weeks, but there was no difference in the incidence of infection in the later stages^18^. Nevertheless, this study does not clearly reveal the mechanism by which infection occurs. Insertion of the chemoport through the left internal jugular vein requires a longer silicone catheter than insertion through the right internal jugular vein because it must pass through the left innominate vein. We speculated that a longer catheter length might increase the risk of infection. No difference in catheter-related thrombosis was observed between the two groups; however, pulmonary embolism occurred more frequently in the infected group (3 [3.8%] vs. 19 [1.2%]; p = 0.048), and the risk ratio was 3.393 (95% CI 1.069–10.765). Therefore, the evaluation of pulmonary embolism can be considered in patients with chemoport-related infections.

Our study results were similar to those of other studies. Infection rates can be reduced through appropriate preventive strategies and chemoport management by well-trained nurses. In this study, we recommend the use of the right internal jugular vein rather than the left internal jugular vein when performing a chemoport procedure to reduce subsequent infection. In addition, the evaluation of the risk of pulmonary embolism in infected patients should be considered.

## Data Availability

The datasets used and/or analyzed during the current study available from the corresponding author on reasonable request.
